# First responder resuscitation teams in a rural Norwegian community: sustainability and self-reports of meaningfulness, stress and mastering

**DOI:** 10.1186/1757-7241-18-25

**Published:** 2010-05-04

**Authors:** Sverre Rørtveit, Eivind Meland

**Affiliations:** 1 Municipal Health Services of Austevoll commune, 5399 Bekkjarvik, Norway; 2 Department of Public Health and Prim Health Care, Section for General Practice, Kalfarveien 31, 5018 Bergen, Norway

## Abstract

**Background:**

Training of lay first responder personnel situated closer to the potential victims than medical professionals is a strategy potentially capable of shortening the interval between collapse and start of cardiopulmonary resuscitation (CPR) in cases of out-of-hospital cardiac arrest. In this study we trained lay first responders personnel in basic life support (BLS) and defibrillation for cases of cardiac arrest and suspected acute myocardial infarction (AMI).

**Methods:**

Forty-two lay first responders living in remote areas or working in industries in the island community of Austevoll, Western Norway, were trained in CPR and defibrillation. We placed particular emphasis on the first responders being able to defibrillate a primary ventricular fibrillation (PVF) in patients with AMI. The trainees were organised in four teams to attend victims of AMI and cardiac arrest while awaiting the arrival of the community emergency medical services. The purpose of the study was to find out whether the teams were able to function during the five-year study project, and to examine whether lives could be saved. The first responders completed questionnaires each year on their experiences of participation. Data on the medical actions of the teams were also collected.

**Results:**

By the end of the project all groups were functioning. The questionnaires evidenced a reasonable degree of motivation and self-evaluated competence in both types of group organisation, but in spite of this attrition effects in the first responders were considerable. The first responders were called out on 24 occasions, for a total of 17 patients. During the study period no case of PVF occurred after the arrival of the first responders, and the number of AMIs was very low, strongly deviating from what was anticipated. No lives were saved by the project.

**Conclusions:**

The teams were sustained for almost five years without any significant deterioration of self-reported stress or mastering, but still showed attrition effects. Evaluated as a medical project the intervention was not successful, but the small scale prevents us from drawing firm conclusions on this aspect.

## Introduction

The odds of surviving a cardiac arrest remain low, and have not improved in the last 20 years, despite the development of new methods in Advanced Life Support (ALS) [1]. The time interval from collapse in cardiac arrest to the start of cardiopulmonary resuscitation (CPR) remains the main determinant of the chance of survival. Training of lay first responder personnel situated closer than medical professionals to the potential victims is a strategy potentially capable of shortening this interval. Worldwide, this has been attempted either by introducing public access defibrillation (PAD) schemes, or by training fire department or police personnel as first responders.

In remote rural settings where PAD schemes are not a practicable option and there are no local full-time fire or police personnel, a strategy of getting first responders to a patient at risk of ventricular fibrillation might be potentially fruitful. A substantial proportion of victims dying from acute myocardial infarction (AMI) die from primary ventricular fibrillation (PVF) shortly after the start of symptoms [2]. If personnel equipped with an automated external defibrillator (AED) and competent in defibrillation and Basic Life Support (BLS) are present at the scene when the patient begins to fibrillate, defibrillation can take place immediately, and the chances of survival are substantially higher [3]. In this study first responder personnel were trained not only in BLS and defibrillation for cases of cardiac arrest, but also and principally for cases where the doctor suspected patients of having AMI.

Our aim was to investigate whether organising first responder personnel in teams was feasible and sustainable over a long time period. Our goal was also to examine the extent to which members of such teams report mental stress, their experience of mastering and to what extent participants felt their tasks to be meaningful. Finally, we wished to investigate whether lives could be saved by the project.

## Materials and methods

The municipality of Austevoll in Western Norway consists of several inhabited islands with a total population of 4400. There is no bridge connection to the mainland. The islands of Hundvåkøy and Storakalsøy have 700 inhabitants. The two islands are connected by a bridge, but at the time of the study period they were not connected by bridge to the main islands of the community. Doctor and ambulance emergency calls to these islands were by ambulance boat and taxi. In 2002 local initiators cooperated with a local supplier of medical equipment, who was also a BLS and defibrillation instructor, and with the municipality medical officer (project leader, SR) to set up first responder teams. The same was also done at two centres of industry in the community, with a total of 150 employees.

For each of the two islands one AED was deployed (neighbourhood teams), along with one AED for each of the industrial areas (workplace teams). In the four teams a total of 42 persons were given a course in BLS combined with defibrillation training, developed by the Norwegian Resuscitation Council. Participation in the first responder teams was on a volunteer basis without remuneration. Of the 42 participants, 39 consented to give personal information: 14 female and 4 male participators in the neighbourhood teams, and 8 and 13 in the workplace teams. In the neighbourhood teams 5 were aged 20-39 and 13 were aged 40-59; the corresponding figures for the workplace teams were 12 and 9.

The project leader took part in the organising, training and surveillance of the teams, and issued the delegations to operate the defibrillator.

The AEDs were placed at dedicated locations on each of the two islands. In an emergency call with suspected AMI or cardiac arrest, the doctor on duty in the municipality would decide whether to alert the first responder team. The first responders did not participate by duty roster, but were called according to a telephone list, with the main emphasis on mobile phones. Two or three first responders were sent to the patient, bringing the AED, with as short a dispatch interval as possible.

The first responders were taught to attach the defibrillating electrodes to the patient's chest, but not to turn the AED on, except in the case of cardiac arrest. The doctor and ambulance personnel would take over the management of the patient on arrival. At the industrial centres, company internal warning systems alerted the first responders.

After the end of every action, the project leader completed a registration form following a telephone conversation with the team members. Recorded events were time point and time intervals of falling ill, telephone calls, response time for the first responders and medical personnel, emergency medical measures taken, and medical end points. Time points and time intervals were estimated by the project leader from the information given by the first responders, often as a mean of the evaluation of the team members, and the recorded intervals of the ambulance personnel were often included in the estimates.

During the study period the neighbourhood groups had considerably more follow-up than the workplace groups. Both types of groups underwent retraining and redelegation once a year. In addition, the neighbourhood groups had a total of eight follow-up meetings originating in a need for evaluation of recent actions, discussions of procedures, and preparing and performing larger-scale training.

Before the start of the project, the members of the first responder teams were asked to give information on their background and their expectations of participation in the project (14 questions). In addition, they were asked to consent to give information on similar topics during the course of the study. Members who gave such consent were sent a questionnaire comprising 15 questions during the study period, and were asked to select the most appropriate answer preformulated on the form. The first questionnaire was sent out six months after the start of the study, and thereafter annually. Participants selected responses on a scale with four levels ranging from 'very good' to 'poor'. A few of the questions had other specific response alternatives according to the nature of the question, all of them graded in four levels.

We estimated the expected AMI and cardiac arrest events from the Norwegian mean incidence of AMI (1997-2001) and a national expert estimate of cardiac arrest [4,5]. From the estimation eleven cases of AMI and four cases of cardiac arrest were anticipated during the planned study period of five years. The results of this paper are from the period May 2002 to May 2007.

### Ethics

The study was approved by the Regional Committee for Medical and Health Research Ethics (REK) and the Norwegian Data Inspectorate.

## Results

### Participants

At the start of the project, the first responder teams comprised 42 members. Twenty-three were organised by the workplace groups and 19 belonged to the neighbourhood groups. At the end of the project, 27 members were still participating, 17 in the workplace groups and 10 in the neighbourhood groups. Table 1 shows the variation in the number of participants over the study period. At the end of the project, one of the two neighbourhood groups had lost five of its original eight members, and its functioning was maintained only by the recruitment of one additional first responder during the study period. Four of the member withdrawals occurred in the last months of the project, between the time of the participants' returning of the last questionnaire and the end of the project.

**Table 1:** Participants by group and time in the project. Response rate at relevant times.

	0.5 years				1.5 years		2.5 years		3.5 years		4.5 years		5 years		
	**M**	**F**	**T**	**A** **N (%)**	**T**	**A** **N (%)**	**T**	**A** **N (%)**	**T**	**A** **N (%)**	**T**	**A** **N (%)**	**M**	**F**	**T**
Workplace	16	7	23	19 (83)	21	17(81)	23	17(74)	19	15 (79)	19	13(68)	13	6	19
Neighb.	4	15	19	18 (95)	18	16(89)	16	14(88)	16	14 (88)	15	13 (87)	2	9	11

Total	20	22	42	37 (88)	39	33(85)	39	31(79)	35	29 (83)	34	26 (76)	15	15	30

M = male, F = female, T = total, A = Answers

### Qestionnaire responses

Thirty-nine of the original participants had consented to complete the questionnaires. Thirty-nine questionnaires were completed and returned at the start of the study; at six months 37 were completed; at two and a half years 31 were completed, and at four and a half years 26 questionnaires were completed. In table 2 we give results from the completed questionnaires by 1/2, 2 1/2 and 4 1/2 years. Throughout the study period the first responders of the neighbourhood groups evaluated their CPR and defibrillation competence, as well as the performance of the group they belonged to, as slightly higher than the workplace groups, but none of these differences are statistical significant. The mean difference between the groups and within each group over time concerning the the other self-reported variables was small and of no clinical relevance. Being in actions was not self-evaluated as obviously changing the first responders' enthusiasm of participation in the project.

**Table 2:** First responders' selfreporting after 1/2, 2 1/2 and 4 1/2 years. Mean values. Values 1 (minimal) - 4 (maximal). Wg = workplace groups, Ng = Neighbourhood groups.

	1/2 year		2 1/2 years		4 1/2 years	
	**Wg (N = 19)**	**Ng** **(N = 18)**	**Wg (N = 17)**	**Ng (N = 14)**	**Wg (N = 13)**	**Ng (N = 12)**

Physical health	3.3	3.7	3.2	3.0	3.5	3.3
Mental health	3.6	3.7	3.5	3.7	3.8	3.7
General anxiety	3.7	3.8	3.5	3.8	3.5	3.7
Health anxiety	3.6	3.7	3.6	3.4	3.5	3.6
Meaningfulness of task	3.6	3.4	3.5	3.6	3.2	3.4
Personal stress	1.5	1.6	1.3	1.7	1.3	1.8
Familiar stress	1.1	1.3	1.0	1.3	1.0	1.3
Sense of group performance	2.8	3.2	2.8	3.3	2.8	3.2
Training in between sessions	2.2	2.3	2.2	2.0	1.9	2.1
Selfrated mastering of CPR and defibrillation	2.9	3.1	2.9	3.4	2.8	3.2
Participated in action^1^	N = 1	N = 6	N = 0	N = 6	N = 1	N = 5
Selfrated performance of action	3	3.7	-	2.3	3	3.8
Change of enthusiasm after action^2^	2.0	2.2	-	2.7	2.0	2.0

^1 ^Number of first responders who had participated in action since last return of questionnaire

^2^Values of variable: 1 = less enthusiastic 2 = unchanged 3 = more enthusiastic 4 = much more enthusiastic

### Patients

The neighbourhood groups were called out on 24 occasions, for a total of 17 patients. On one occasion the group should have been alerted according to procedure, but failure of communication prevented this, and this case is not included in the material. The patients were aged 36-92 years, with a mean of 66 years. Since more than one first responder took part in each action, a total of 63 person-actions are recorded for the neighbourhood members. Seven first responders participated in cardiopulmonary resuscitation, one of them by giving defibrillation. The mean participation per neighbourhood team member per year was 0.74 actions.

The reason for call out was cardiac arrest in 6 of the 24 actions, and suspected AMI in 18 cases. For the arrest patients, the indication "no shock indication" was given by the first responder's turning on the automated defibrillator in five of the six cases. This means the initial rhythm was asystole, as this was the way the machines were programmed from the manufacturer. To the sixth patient the machine was not turned on, since there was a verified too long interval without circulation or CPR. All the six cardiac arrest patients were declared dead on scene by the doctor. In two instances, by chance the first personnel to reach the patient was a paramedic or doctor.

The time intervals from alarm call to arrival of first responder and of AED, is given for suspected AMI and for cardiac arrest in table 3. The first responder arrived at the patient in a median of 22.5 minutes before the ambulance personnel and doctor; the AED was there in a median of 20 minutes before the ambulance and doctor arrived.

**Table 3:** Response times (interval of minutes)

	Suspected AMI		Cardiac arrest	
	**Call-first responder^1^**	**Call-AED^2^**	**Call-first responder**	**Call-AED**

0-2 min	2	-	-	-
3-5 min	-	-	2	1
9-10 min	4	4	-	-
11-15 min	5	4	4	2
16-20 min	3	2	-	1
21-25 min	-	2	-	1
26-40 min	3	4	-	-
41-60	1	1	-	-
AED not brought	-	-	-	1
Unknown	-	1	-	-

Total	18	18	6	6

^1 ^Interval from alarm call to arrival of the first responder at the patient

^2 ^Interval from alarm call to arrival of automated defibrillator

In the 18 cases of suspected AMI, acute chest pain was the first symptom in 13 cases and other AMI-related symptoms in five cases. Upon doctor's examination on site, the condition was deemed not to necessitate hospitalisation in four cases. Of the remaining cases, one patient was confirmed in hospital as having an AMI, twelve were evaluated not to have AMI, and for one patient hospital data was not obtained. None of the patients with suspected AMI as the reason for the emergency call out had a cardiac arrest while attended only by the first responders.

### First responders' actions

In 62% of alerts the first team member called was able to attend the patient, and in 70% the second team member called was able to respond. In one instance the ambulance personnel were not able to contact any of the first responders. The workplace groups were never called into action during the study period. Individual members of the workplace groups did take part in emergencies, attending an AMI-suspected patient or performing CPR on four occasions, though all of these instances were outside the workplace.

The training procedure of attaching the defibrillator electrodes to the patient's chest upon the first responder's arrival at the patient was followed in five instances and not followed in 12 instances. Reported reasons for not attaching the electrodes were evaluation of the situation by the first responders as non-urgent (2 instances), not wanting to risk the integrity of the patient (1), instructions by the paramedic or doctor not to attach the electrodes (1), and when the ambulance and doctor would arrive very shortly after the first responder (2). In five instances no reason was given for deviating from the procedure, and in one instance it is not known whether the electrodes were attached or not.

## Discussion

Deployment of automated external defibrillators and training of first responders is carried out worldwide ranging in scale from very large projects like the US Public Access Defibrillation Trial [6] to very small schemes with participation of only a few people.

Our study gives data on the formation and follow-up of first responders organised in four groups of just under ten persons per group. We give data on the opinions, experiences, medical activity and participation of each member of the groups, collected annually for a time span of five years. This study thus documents the feasibility and sustainability of first responder groups. We maintain that such documentation should be mandatory in these types of projects. To our knowledge, this kind of information has not been published by others.

All four groups were functioning at the end of the five-year period. Over the study period the participants generally reported a reasonable, though not high level of self-evaluated competence in CPR and defibrillation, a high degree of meaningfulness of the participation, and low self-rated stress. In spite of a high difference in follow-up and experience of actions between the two types of group organisation, their subjectively evaluated meaningfulness and competence of CPR and defibrillation was surprisingly similar.

Four of the member withdrawals occurred in the last months of the project. It is possible that this was due to a reduction in perceived meaningfulness or increased sense of stress not captured by the last round of questionnaires, which were performed a half year before the end of the project. Some of the members of the neighbourhood group with the highest withdrawal said they felt the task was burdensome, since responsibility was shared among only a few people. We evaluate the high withdrawal in the end period of the project as a real effect of wear and tear, and it was seen in the groups exposed to real actions.

The dispatch procedure resulted in overlong response times, and adherence to the training procedure was suboptimal. The findings of this study imply that medical professionals organising first responder schemes should consider thoroughly which practical circumstances ought to be present for a project to be successful. The possibility that the first responders will stay the course should be discussed with participants from the beginning. At the yearly retraining and delegation, we recommend holding an informal discussion with the first responder group, with emphasis on how the group members feel about their participation. If a first responder project is no longer functioning in practice, it should be formally terminated.

This study was in part stimulated by the idea of getting defibrillation-competent personnel rapidly to patients with suspected AMI, in order to defibrillate them in case of PVF [3]. During the study period no case of PVF occurred after the arrival of the first responders, and in fact the number of AMIs was extremely low, strongly deviating from what was anticipated. This type of discrepancy is possible in a small study population such as ours. Shifting AMI epidemiology, with fewer ST-elevation AMIs and an older AMI population, may also have contributed to the results [7].

The Norwegian Air Ambulance 'Early Heartstart' scheme deployed AEDs and have reported some of their data[8]. It was initiated in 2002 and summarised in annual reports, the most recent for 2005. In their project, 228 AEDs were deployed by the end of 2005 to 181 Norwegian municipalities, primarily to fire departments. In 2005 the AEDs were operated in 44 medical emergencies, and in 42% of these defibrillation was performed. Two of the defibrillated patients survived. Diagnoses for the actions and a list of the medical procedures undertaken are not given, and the methods of data collection are not described. Planned procedures for the first responders' medical actions and their cooperation with the municipality doctors and paramedics are similarly not included.

It is well known that other organisations and companies cooperate with suppliers of AEDs in training CPR and defibrillation and deploying AEDs at workplaces and public locations. We have not been able to obtain data from any of these. A main problem concerning the widespread organisation of first responder groups is that the extent and reliability of the data reporting are highly variable, and for most probably non-existent. This means that we do not know in the majority of cases whether defibrillation first responder projects save lives.

In conclusion, we judge the validity of the study to be satisfactory, as we carefully observed and registered data during the study period according to a preplanned procedure. All groups functioned throughout the five-year study period. No life-saving effect was detected during this project, but this should be seen in the context that the main weakness of the study is the low population of potential patients and therefore low power to determine any real life-saving effects.

## Competing interests

The authors declare that they have no competing interests.

## Authors' contributions

SR initiated the study and collected the data, worked out the first draft of the manuscript, took part in the design of the study and the statistical analyses. EM took part in the design of the study and the statistical analyses, and has revised the manuscript. Both authors read and approved the final manuscript.
